# Swelling and Drug Release in Polymers through the Theory of Poisson–Kac Stochastic Processes

**DOI:** 10.3390/gels7010032

**Published:** 2021-03-22

**Authors:** Alessandra Adrover, Claudia Venditti, Massimiliano Giona

**Affiliations:** Dipartimento di Ingegneria Chimica, Materiali e Ambiente, Sapienza Università di Roma, via Eudossiana 18, 00184 Roma, Italy; claudia.venditti@uniroma1.it (C.V.); massimiliano.giona@uniroma1.it (M.G.)

**Keywords:** swelling, non-Fickian transport, drug release, Poisson–Kac processes, moving-boundary models

## Abstract

Experiments on swelling and solute transport in polymeric systems clearly indicate that the classical parabolic models fail to predict typical non-Fickian features of sorption kinetics. The formulation of moving-boundary transport models for solvent penetration and drug release in swelling polymeric systems is addressed hereby employing the theory of Poisson–Kac stochastic processes possessing finite propagation velocity. The hyperbolic continuous equations deriving from Poisson–Kac processes are extended to include the description of the temporal evolution of both the Glass–Gel and the Gel–Solvent interfaces. The influence of polymer relaxation time on sorption curves and drug release kinetics is addressed in detail.

## 1. Introduction

Many fluids and specifically polymeric liquids, suspensions, and gels possess viscoelastic properties, which, in the simplest case, can be characterized through a single relaxation time [1], leading to constitutive equations with memory for the stress tensor as a function of the deformation tensor. Since momentum transport is affected by changes in the constitutive equations, it is natural to assume that the memory effects in viscoelastic materials impact on the transport properties and the associated phenomena, such as glassy-rubbery transition, swelling, and the release of a solute dispersed in the material. Indeed, experiments on swelling and solute transport in polymeric systems indicate that the classical parabolic models (in which the mass flux is proportional to the concentration gradient) fail to predict typical and distinguishing features, such as Case II diffusion and concentration overshoot in sorption experiments [2,3]. A systematic analysis of non-Fickian sorption kinetics in polymer films can be found in Sanopoulou and Petropoulos, 2001 [4], where a list, from 1 to 6, of characteristic deviations from Fickian sorption behaviour is presented.

From the experimental point of view, the effect of polymer relaxation on sorption curves can be investigated in many different ways. A couple of examples among the many present in the literature are discussed here. Focusing on hydrogels, stress relaxation experiments can be carried out to determine the time scale of macromolecular adjustments during the swelling process. Peppas and Brazel [5] measured the characteristic time for stress relaxation in a swollen polymer when subjected to a mechanical stress by the Instron and then related this time to the time required for a dry polymer to adjust to being placed in a solvent, assuming one-dimensional transport. These authors observed that the stress relaxation time constants at 37 °C for redox- and thermally initiated free radical solution polymerized samples of P(HEMA-co-MMA) crosslinked by EGDMA and glutaraldehyde-crosslinked PVA samples changes significantly with the amount of cross-linking agent and PVA molecular weight. Therefore, it is possible to design different materials and different experiments to evaluate the contribution of polymer relaxation to the experimental sorption curves. Focusing on organic vapor sorption in polymer film, Sanopoulou and Petropoulos [4] proposed different diagnostic criteria for the physical origin of non-Fickian kinetic features, some strictly related to the presence of viscous relaxation phenomena. Interval sorption as well as integral sorption experiments on films with different thicknesses can be useful to identify the role and contribution of polymer relaxation. By considering that the Deborah number is inversely proportional to the film thickness squared, these authors analyzed the absorption kinetics of CA-Acetone systems and observed significant differences between sorption curves when changing the film thickness from 42 to 112 μm, thus decreasing the Deborah number by a factor of 10.

Form the theoretical point of view, a first coherent approach in this direction was undertaken by Cattaneo [6,7] in order to solve some paradoxes in the parabolic theory of heat transfer. The original articles by Cattaneo deal with the heat transfer (so that the final result of this investigation is usually referred to as the *Cattaneo heat equation*), but the extension to mass transport is straightforward. Cattaneo’s model is indeed a hyperbolic model in which the diffusive flux satisfies a constitutive equation identical to the constitutive equations used in linear viscoelasticity.

As Fickian diffusion corresponds microscopically to a stochastic motion described in terms of Wiener processes, an important conceptual issue is the determination of the kinematic equations underlying the Cattaneo equation. In other words, which stochastic model for particle transport determines macroscopically the concentration field representing the solution of the Cattaneo equation. The ultimate answer to this fundamental question has been given by Mark Kac, at least for one-dimensional spatial problems [8], and his approach provides not only a simple kinematic model associated with the occurrence of memory effects in transport equations, but also an alternative, and physically significant way of describing transport processes via the concept of partial densities (or partial concentrations). Both the Cattaneo hyperbolic transport theory and the Kac stochastic model played a fundamental role in developing more refined theories for non-equilibrium processes, and their stochastic characterization. Cattaneo’s model represented the cornerstone in the development of the Extended Thermodynamics proposed by Müller and Ruggeri [9,10] to generalize the classical theory of irreversible processes, and the Kac model is the starting point in the development of a wide class of stochastic processes possessing Markovian transitions, referred to as Generalized Poisson–Kac processes [11,12,13].

The variable surface concentration model proposed by Long and Richman as well as the diffusion-relaxation model by Berens and Hopfenberg (see [14] for a detailed review of these two classical models) represent the first attempts to describe the sorption process in glassy polymers as a linear superposition of phenomenologically independent contributions from Fickian diffusion and polymeric relaxation, the latter influencing the sorptive capacity of the polymer. The extension to more than one distinct structural relaxation process with different relaxation time has been also proposed [15,16] within the framework of a purely Fickian (diffusional) solvent transport with fixed boundaries.

A significant step ahead in the field is represented by the work by Cohen and White [17], in which the major effect of a diffusing penetrant on the polymer entanglement network is taken to be the inducement of a differential viscoelastic stress. This couples diffusive and mechanical processes through a viscoelastic response, where the strain depends upon the amount of penetrant. Subsequently, many authors, e.g., Grassi et al. [18,19] first, and Wu and Brasel [20] later, following Camera-Roda and Sarti [21,22] and Cohen [17], considered a solvent flux, different from the simple Fick’s law, to account for polymer relaxation in mass transport, thus arriving at a hyperbolic formulation of the transport problem. However, in these works, the effect of the moving boundaries—namely, the Glass–Gel and the Gel–Solvent interfaces—has been neglected as well as the convective contribution to mass transport induced by the presence of a swelling velocity. The “swelling flux” has been accounted for in the model proposed by Lamberti et al. [23,24], but only very low values of the Deborah number have been considered, thus disregarding the effect of polymer relaxation time. In this article, the Poisson–Kac (PK) stochastic approach is adopted and extended to describe and investigate the effect of polymer relaxation time on solvent penetration in glassy polymers. To this end, an extra convective term is included in the classical partial wave transport equations to account for the contribution of the swelling point-wise velocity. Indeed, the model accounts for the three contributions to solvent transport—namely, the diffusive flux, the swelling convective flux, and the relaxation flux. The movements of the two fronts, the Gel-Solvent and the Glass-Gel interfaces, are also accounted for and described in terms of PK partial waves. This enables (I) to study the influence of the Deborah number (from low to high values) on the sorption curves and (ii) to compare the results of the hyperbolic and the classical parabolic transport schemes, accounting for both swelling and moving boundaries.

The original PK model has been also generalized to define a broader class of processes possessing Markovian transitions, where the characteristic velocity and the transition rate are continuous functions of the overall density [13].

This extension permits the investigation of the effect of both a polymer relaxation time and a solvent diffusivity that are exponential functions of the local solvent concentration, as established by simplified versions of the free volume theory [25,26,27]. In particular, the introduction of a non-constant polymer relaxation time leads to interesting and unexpected behaviours of the sorption curves, due to the presence of a nonlinear convective term that facilitates solvent penetration and speeds up the outward movement of the Gel-Solvent interface.

In the paper, we also investigate the influence of the polymer relaxation time on the release kinetics of a drug initially loaded in the thin dry film. Release kinetics are strongly influenced by the polymer relaxation time and, unexpectedly, are not necessarily slowed down for intermediate values of the Deborah number.

## 2. Poisson–Kac Processes

The counterpart of a linear viscoelastic constitutive equation in mass transport is represented by the Cattaneo equation. Let c(x,t) be the solvent concentration in a polymer-solvent solution and $J_{c}\left(x,t\right)$ its diffusive flux. We refer here to the concept of “diffusive flux” as the flux associated with pure random molecular motion in the absence of any external macroscopic drift. Below, one-dimensional spatial problems are considered for the sake of simplicity.

In the absence of sources, the balance equation for c(x,t) reads:

(1) $$\frac{\partial c(x,t)}{\partial t}\;=\;-\frac{\partial J_{c}\left(x,t\right)}{\partial x}$$

In the classical Fickian transport theory, the constitutive equation for the flux is:

(2) $$J_{c}\left(x,t\right)\;=\;-D\;\frac{\partial c(x,t)}{\partial x}$$

In Cattaneo theory, the constitutive equation for the flux $J_{c}\left(x,t\right)$ takes the form:

(3) $$t_{r}\;\frac{\partial J_{c}\left(x,t\right)}{\partial t}\;+\;J_{c}\left(x,t\right)\;=\;-D\;\frac{\partial c(x,t)}{\partial x}$$

where *D* is a diffusivity and $t_{r}$ is the characteristic relaxation time. For $t_{r}\;=\;0$, Equation (3) reduces to the classical Fickian constitutive equation, in which the flux is proportional to the concentration gradient.

The introduction of a constitutive equation with memory, such as Equation (3), determines major and significant changes in the nature of the diffusive propagation. To begin with, from Equations (1)–(3), it follows that the evolution equation for the concentration c(x,t) attains the form:

(4) $$t_{r}\;\frac{\partial^{2}c\left(x,t\right)}{\partial t^{2}}\;+\;\frac{\partial c(x,t)}{\partial t}\;=\;D\;\frac{\partial^{2}c\left(x,t\right)}{\partial x^{2}}$$

In other words, it attains a hyperbolic character due to the second-order derivatives with respect to time. This corresponds to the evolution of solvent concentration in the form of waves with dispersion, possessing the characteristic propagation velocity $\sqrt{D/t_{r}}$.

For any problem in mass transport involving moving molecules and particles, it is of the highest conceptual and practical interest to determine the structure of the microscopic particle kinematics that originate the emergent macroscopic behavior for the concentration field. Brownian motion, expressed with regard to the long-term properties by Wiener processes, represents the kinematics of Fickian diffusion. For the hyperbolic transport Equation (4), M. Kac showed that the one-dimensional Cattaneo transport equation is originated from the microscopic particle motion described by the following kinematic equation [8]:

(5) $$\frac{dx(t)}{dt}\;=\;b_{0}\;{\left(-1\right)}^{\chi (t,\lambda )}$$

where $b_{0}\;>\;0$ is a constant possessing the dimension of a velocity and χ(t,λ) is a Poisson counting process characterized by the transition rate λ > 0. The Poisson process χ(t,λ) attains values 0,1,2,… spanning the natural numbers. Meanwhile, in the classical applications of probability theory, Prob[χ(0,λ) = 0] = 1, with reference to Equation (5) it is more convenient to consider the following initial conditions Prob[χ(0,λ) = 0] = Prob[χ(0,λ) = 1] = 1/2, Prob[χ(0,λ) > 1] = 0, in order to avoid any initial biasing effects. From Equation (5), it follows that the trajectories associated with this kinematic law correspond to the continuous union of straight segments, with slopes of either $+b_{0}$ or $-b_{0}$. The transition time θ between two consecutive velocity switches is controlled by the parity of ${\left(-1\right)}^{\chi (t,\lambda )}$ is a random variable characterized by an exponential probability density function $p_{\theta }\left(\theta \right)\;=\;\lambda \;e^{-\lambda \;\theta }$.

The process X(t), the realization of which is x(t) defined by the kinematics Equation (5), is not Markovian, and this feature determines the occurrence of memory effects in the flux of constitutive equations; Equation (3). Nevertheless, it is still possible to construct a Markovian embedding for X(t) by considering the joint stochastic process (X(t),S(t)), where $S\left(t\right)\;=\;{\left(-1\right)}^{\chi (t,\lambda )}$ is the parity function attaining values ±1, and determining the velocity direction. Since S(t) is a binary process attaining only two values, it is possible to introduce the two partial probability densities $p_{\pm }\left(x,t\right)$ where:

(6) $$p_{\pm }\left(x,t\right)\;dx\;=\;\mathrm{Prob}\left[X\left(t\right)\;\in \;\left(x,x\;+\;dx\right),\;{\left(-1\right)}^{\chi (t,\lambda )}\;=\;\pm 1\right]$$

Additionally, the application of the Chapman–Kolmogorov equation to the embedding process (X(t),S(t)) provides the following evolution equations for $p_{\pm }\left(x,t\right)$:

(7) $$\begin{array}{ccc} \frac{\partial p_{+}\left(x,t\right)}{\partial t} & \;=\; & -b_{0}\;\frac{\partial p_{+}\left(x,t\right)}{\partial x}\;-\;\lambda \left[p_{+}\left(x,t\right)\;-\;p_{-}\left(x,t\right)\right] \end{array}$$

(8) $$\begin{array}{ccc} \frac{\partial p_{-}\left(x,t\right)}{\partial t} & \;=\; & b_{0}\;\frac{\partial p_{-}\left(x,t\right)}{\partial x}\;+\;\lambda \left[p_{+}\left(x,t\right)\;-\;p_{-}\left(x,t\right)\right] \end{array}$$

These correspond to a system of two first-order linear partial differential equations. Given the partial densities $p_{\pm }\left(x,t\right)$, also referred to as partial waves, the overall probability density function for X(t) is expressed by:

(9) $$p\left(x,t\right)\;=\;p_{+}\left(x,t\right)\;+\;p_{-}\left(x,t\right)$$

The associated probability flux $J_{p}\left(x,t\right)$ takes the form:

(10) $$J_{p}\left(x,t\right)\;=\;b_{0}\;\left[p_{+}\left(x,t\right)\;-\;p_{-}\left(x,t\right)\right]$$

A probabilistic terminology is here adopted for describing the statistical properties of an ensemble of particles. That is to say, once applied to a mass transport problem, the mass/molar concentration c(x,t) is related to the density function p(x,t), by a proportionality relation, c(x,t) = Kp(x,t), where the constant *K* admits the dimension of a mass/number-of-moles, depending on the physical meaning of c(x,t).

The use of the partial densities provides a very clear statistical understanding on the evolution of particle concentrations associated with the kinematics (5). The evolution of the overall density p(x,t) can be decomposed into the superposition of two partial waves $p_{\pm }\left(x,t\right)$ propagating at constant velocity in the two opposite directions along the *x*-axis, and mutually recombining at the rate λ which defines the statistics of the transition times of the Poisson process. For this reason, the stochastic process (5) can be defined as the elementary Poisson–Kac process.

By summing Equations (7) and (8), the equation for the overall density p(x,t) follows thus:

(11) $$\frac{\partial p(x,t)}{\partial t}\;=\;-\frac{\partial J_{p}\left(x,t\right)}{\partial x}$$

Meanwhile, the constitutive equation for the probability flux $J_{p}\left(x,t\right)$, defined by Equation (10), is easily obtained by taking the difference between the two Equations (7) and (8) multiplied by $b_{0}$:

(12) $$\frac{\partial J_{p}\left(x,t\right)}{\partial t}\;=\;-b_{0}^{2}\;\frac{\partial p(x,t)}{\partial x}\;-\;2\;\lambda \;J_{p}\left(x,t\right)$$

Equation (12) coincides with the Cattaneo constitutive Equation (3) upon the identification of the relaxation time $t_{r}$ and the diffusivity *D* as:

(13) $$t_{r}\;=\;\frac{1}{2\;\lambda }\;,\;D\;=\;\frac{b_{0}^{2}}{2\;\lambda }$$

The original Poisson–Kac model can be generalized to define a broader class of processes possessing Markovian transitions, and referred to as Generalized Poisson–Kac processes [11,12]. For the scope of the present article, it is interesting to consider a nonlinear expansion of the model where both the characteristic velocity $b_{0}$ and the transition rate λ are continuous functions of the overall density p(x,t):

(14) $$b_{0}\;=\;{\bar{b}}_{0}\;\beta \left(p\right)\;,\;\lambda \;=\;{\bar{\lambda }}_{0}\;\ell \left(p\right)$$

In this case, the balance equations for the partial densities $p_{\pm }\left(x,t\right)$ are altogether similar to Equations (7) and (8):

(15) $$\begin{array}{ccc} \frac{\partial p_{+}}{\partial t} & \;=\; & -{\bar{b}}_{0}\;\frac{\partial [\beta \left(p\right)\;p_{+}]}{\partial x}\;-\;{\bar{\lambda }}_{0}\;\ell \left(p\right)\left[p_{+}\;-\;p_{-}\right] \end{array}$$

(16) $$\begin{array}{ccc} \frac{\partial p_{-}}{\partial t} & \;=\; & {\bar{b}}_{0}\;\frac{\partial [\beta \left(p\right)\;p_{-}]}{\partial x}\;+\;{\bar{\lambda }}_{0}\;\ell \left(p\right)\left[p_{+}\;-\;p_{-}\right] \end{array}$$

The equation for p(x,t) follows by summing Equations (15) and (16):

(17) $$\frac{\partial p}{\partial t}\;=\;-\frac{\partial [\beta \left(p\right)\;\psi_{p}]}{\partial x},\;\psi_{p}\left(x,t\right)\;=\;{\bar{b}}_{0}\left[p_{+}\;-\;p_{-}\right]$$

Observe that, due to the nonlinearities, the density flux is given by:

(18) $$J_{p}\left(x,t\right)\;=\;\beta \left(p\right)\;\psi_{p}\left(x,t\right)$$

where $\psi_{p}\left(x,t\right)$ is an auxiliary field. The evolution equation for the auxiliary field $\psi_{p}\left(x,t\right)$ is obtained by taking the difference between the two Equations (15) and (16) multiplied by ${\bar{b}}_{0}$:

(19) $$\frac{\partial \psi_{p}}{\partial t}\;=\;-{\bar{b}}_{0}^{2}\;\frac{\partial [\beta (p)\;p]}{\partial x}\;-\;2\;{\bar{\lambda }}_{0}\ell \left(p\right)\;\psi_{p}$$

From this, the constitutive equation for the flux is recovered by enforcing definition (18). Equation (19) implies that either the relaxation time $t_{r}$ or the diffusivity *D* are functions of the space and time coordinates through their dependence on the overall density p(x,t), namely:

(20) $$t_{r}\left(p\right)\;=\;\frac{1}{2\;{\bar{\lambda }}_{0}}\frac{1}{\ell (p)}\;,\;D\left(p\right)\;=\;\frac{{\bar{b}}_{0}^{2}}{2\;{\bar{\lambda }}_{0}}\frac{\beta^{2}\left(p\right)}{\ell (p)}\;=\;D_{0}\frac{\beta^{2}\left(p\right)}{\ell (p)}$$

However, it is important to observe that, in the Kac limit ${\bar{b}}_{0},{\bar{\lambda }}_{0}\;\to \;\infty$, $\frac{{\bar{b}}_{0}^{2}}{2\;{\bar{\lambda }}_{0}}\;\to \;const$, the equation for p(x,t) is, as expected, a parabolic transport equation:

(21) $$\frac{\partial p}{\partial t}\;=\;D_{0}\;\frac{\partial }{\partial x}\left[\frac{\beta^{2}\left(p\right)}{\ell (p)}\;\frac{\partial p}{\partial x}\right]\;-\;D_{0}\;\frac{\partial }{\partial x}\left[-\beta \left(p\right)\frac{\partial \beta (p)}{\partial p}\frac{\partial p}{\partial x}\;p\right],\;D_{0}\;=\;\frac{{\bar{b}}_{0}^{2}}{2\;{\bar{\lambda }}_{0}}$$

However, a new highly nonlinear convective term appears (the second term in the right-hand side of Equation (21)), that has no counterpart in the corresponding parabolic formulation of a transport model, even accounting for a non-constant concentration-dependent diffusion coefficient.

## 3. Parabolic Transport Model for Case II Diffusion

**Figure 1:**
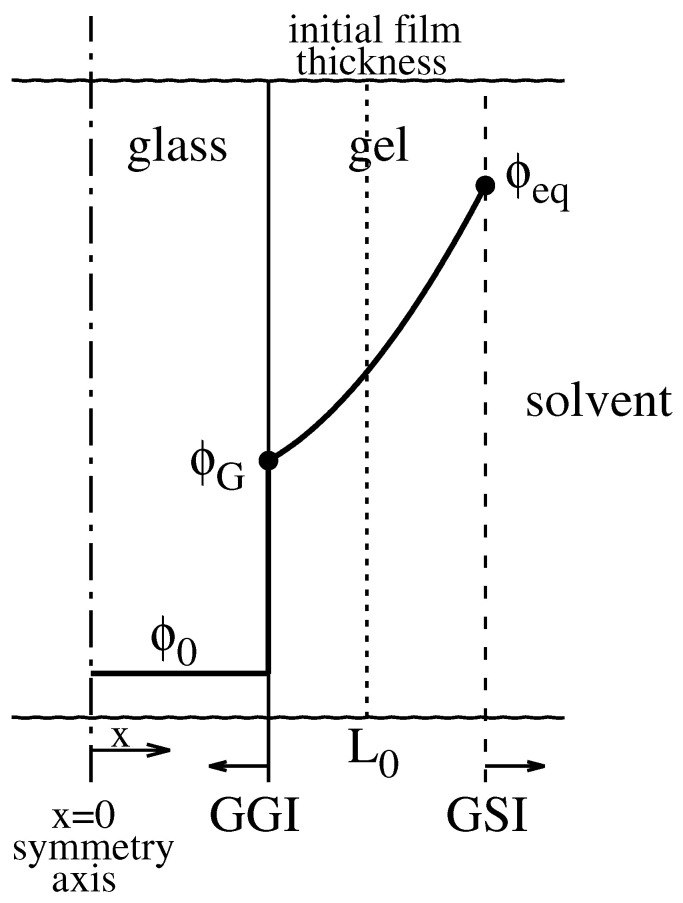
Schematic representation of solvent penetration in a glassy film (1-d Case II diffusion in the *x* direction). $\phi_{0}$, $\phi_{G}$, and $\phi_{eq}$ are the solvent volume fractions in the glassy film, at the Glassy–Gel interface (GGI), and at the Gel–Solvent interface (GSI), respectively.

Swelling results from the solvent penetration into the polymer. The solvent enhances the mobility of polymer chains by converting the glassy matrix into a swollen, rubbery material. Sorption in glassy polymers, usually referred to as Case II diffusion, is typically characterized by two moving fronts: (i) a sharp interface between unpenetrated glass and swollen polymer (Glassy–Gel Interface GGI) that propagates inwards into the film and (ii) a Gel–Solvent Interface (GSI) that, in the absence of dissolution, moves outwards and progressively increases the gel layer thickness. A schematic representation of solvent penetration in a glassy film is depicted in Figure 1.

The classical 1-d transport equations, not accounting for the relaxation time of polymer, describe the solvent penetration and the temporal evolution of the moving interfaces GGI and and GSI in terms of parabolic partial differential equations for the solvent volume fraction ϕ(x,t) in the gel layer:

(22) $$\frac{\partial \phi }{\partial t}\;=\;-\frac{\partial J}{\partial x}\;=\;-\frac{\partial }{\partial x}\left(-D\frac{\partial \phi }{\partial x}\;+\;v_{sw}\;\phi \right)\;=\;\frac{\partial }{\partial x}\left(D\frac{\partial \phi }{\partial x}\;\left(1\;-\;\phi \right)\right),\;GGI\left(t\right)\;<\;x\;<\;GSI\left(t\right),\;t\;>\;0$$

where *D* is the solvent diffusivity and $v_{sw}$ is the point-wise swelling velocity, assumed to be equal (and opposite in sign) to the solvent diffusive (volumetric) flux [28,29,30,31,32,33]:

(23) $$v_{sw}\left(x,t\right)\;=\;D\frac{\partial \phi }{\partial x}$$

The solvent and polymer are assumed to be incompressible and to mix with no volume change [31].

At the Gel–Solvent interface GSI(t), solvent/polymer thermodynamic equilibrium $\phi \;=\;\phi_{eq}$ is assumed, and the temporal evolution of GSI(t) is described by the Stefan equation [31]:

(24) $$\phi \;=\;\phi_{eq},\;\;\frac{d\;GSI}{dt}\;=\;v_{sw}{|}_{GSI(t)}\;\;at\;x\;=\;GSI\left(t\right).$$

On the Glass–Gel front GGI(t), a threshold concentration to initiate swelling $\phi \;=\;\phi_{G}\;>\;\phi_{0}$ is assumed for the solvent [34], with $\phi_{0}$ being the initial solvent volume fractions in the dry film. Correspondingly, the temporal evolution of GGI(t) reads as:

(25) $$\left(\phi_{G}\;-\;\phi_{0}\right)\;\frac{d\;GGI}{dt}{\;=\;J|}_{GGI(t)}\;at\;x\;=\;GGI\left(t\right)$$

When GGI(t) reaches the symmetry axis x = 0, the glassy phase disappears and the zero-flux boundary condition applies: J(0,t) = 0.

The initial conditions for ϕ(x,t), GGI(t), and GSI(t) are $GGI\left(0\right)\;=\;L_{0}\;-\;\epsilon$, $GSI\left(0\right)\;=\;L_{0}$, and $\phi \left(x,0\right)\;=\;\phi_{G}$, with $\epsilon ≃10^{-4}L_{0}$—i.e., it is assumed that a gel layer of infinitesimal thickness is already formed and, consistently, that the solvent volume fraction ϕ(x,0) for $L_{0}\;-\;\epsilon \;<\;x\;<\;L_{0}$ has attained the Glass–Gel threshold value $\phi_{G}$.

Solvent diffusivity *D* in the swollen layer can be assumed constant or an increasing exponential function of the point-wise solvent volume fraction: ϕ [22,35,36]

(26) $$D\left(\phi \right)\;=\;D_{eq}\exp\left(-\gamma \;\frac{\phi \;-\;\phi_{eq}}{\phi_{G}\;-\;\phi_{eq}}\right)\;=\;D_{G}\exp\left(\gamma \;\frac{\phi_{G}\;-\;\phi }{\phi_{G}\;-\;\phi_{eq}}\right),\;\gamma \;>\;0$$

where $D_{G}$ and $D_{eq}\;=\;D_{G}\exp\gamma$ are the minimum and maximum solvent diffusivities at the minimum $\phi_{G}$ and at the maximum $\phi_{eq}$ solvent volume fractions in the gel layer.

The amount of absorbed solvent M(t) (per unit area of the film), at each time instant during the course of the swelling process can be evaluated as:

(27) $$M\left(t\right)\;=\;2\;\rho_{s}\int_{GGI(t)}^{GSI(t)}\phi \left(x^{\prime },t\right)\;dx^{\prime }$$

where $\rho_{s}$ is the solvent mass density. When equilibrium (asymptotic) conditions are reached, the glassy phase completely disappears—i.e., $GGI_{\infty }\;=\;0$—and the half-thickness of the fully swollen film $L_{\infty }\;=\;\;GSI_{\infty }$, in the absence of dissolution, reaches its asymptotic value.

(28) $$\frac{L_{\infty }}{L_{0}}\;=\;\frac{GSI_{\infty }}{GSI_{0}}\;=\;\frac{1\;-\;\phi_{0}}{1\;-\;\phi_{eq}}$$

Correspondingly, the asymptotic amount of absorbed solvent $M_{\infty }$ (per unit area of the film) attains the form:

(29) $$M_{\infty }\;=\;2\;\rho_{s}\int_{0}^{GSI_{\infty }}\phi_{eq}\;dx\;=\;2\;\rho_{s}\;L_{\infty }\;\phi_{eq}\;=\;2\;\rho_{s}\;L_{0}\;\phi_{eq}\;\frac{1\;-\;\phi_{0}}{1\;-\;\phi_{eq}}$$

## 4. Poisson–Kac Transport Model for Case II Diffusion

The main goal is to model the Case II diffusion process by including the effects of polymer relaxation time. This can be addressed through the following steps:

1. identify the solvent volume fraction ϕ(x,t) with the overall probability density function p(x,t) of the Poisson–Kac stochastic process—i.e., $p\left(x,t\right)\;=\;p_{+}\left(x,t\right)\;+\;p_{-}\left(x,t\right)\;=\;\phi \left(x,t\right)$;
2. include in the dynamics of the partial waves $p_{+}\left(x,t\right)$ and $p_{-}\left(x,t\right)$ a further convective contribution accounting for the swelling velocity $v_{sw}\left(x,t\right)$ due to solvent penetration;
3. describe the movement of the Glass–Gel interface and Gel–Solvent interface in terms of partial waves $p_{\pm }\left(x,t\right)$;
4. account for the effect of polymer relaxation time on the boundary condition at the Gel–Solvent interface.

At first, we consider the simplest case of a constant relaxation time $t_{r}$ and a constant diffusivity *D*— i.e., a constant transition rate λ and a constant characteristic velocity $b_{0}$, Equation (13). By considering that (i) the point-wise swelling velocity $v_{sw}\left(x,t\right)$ is assumed to be equal and opposite in sign to the diffusive flux, and that (ii) the “diffusive” flux in the Poisson–Kac formulation is given by Equation (10), it naturally follows that:

(30) $$v_{sw}\left(x,t\right)\;=\;-J_{p}\left(x,t\right)\;=\;-b_{0}\left[p_{+}\left(x,t\right)\;-\;p_{-}\left(x,t\right)\right]$$

The evolution equations for the partial densities $p_{+}\left(x,t\right)$ and $p_{-}\left(x,t\right)$ in the gel layer GGI(t) < x < GSI(t) attain the form:

(31) $$\begin{array}{ccc} \frac{\partial p_{+}\left(x,t\right)}{\partial t} & \;=\; & -b_{0}\;\frac{\partial p_{+}\left(x,t\right)}{\partial x}\;\;-\;\frac{\partial [v_{sw}\left(x,t\right)\;p_{+}\left(x,t\right)]}{\partial x}\;-\;\lambda \left[p_{+}\left(x,t\right)\;-\;p_{-}\left(x,t\right)\right] \end{array}$$

(32) $$\begin{array}{ccc} \frac{\partial p_{-}\left(x,t\right)}{\partial t} & \;=\; & b_{0}\;\frac{\partial p_{-}\left(x,t\right)}{\partial x}\;\;-\;\frac{\partial [v_{sw}\left(x,t\right)\;p_{-}\left(x,t\right)]}{\partial x}\;+\;\lambda \left[p_{+}\left(x,t\right)\;-\;p_{-}\left(x,t\right)\right] \end{array}$$

By introducing the dimensionless time $\tau \;=\;t/t_{r}\;=\;2\lambda t$ and the dimensionless spatial coordinate $z\;=\;x/L_{0}$, the transport equation for the partial waves $p_{\pm }\left(z,\tau \right)$ in the gel layer GGI(τ) < z < GSI(τ) reads as:

(33) $$\begin{array}{ccc} \frac{\partial p_{+}}{\partial \tau } & \;=\; & -\sqrt{De}\;\;\frac{\partial [\left(1\;-\;p_{+}\;+\;p_{-}\right)p_{+}]}{\partial z}\;-\;\frac{1}{2}\left[p_{+}\;-\;p_{-}\right] \end{array}$$

(34) $$\begin{array}{ccc} \frac{\partial p_{-}}{\partial \tau } & \;=\; & \sqrt{De}\;\;\frac{\partial [\left(1\;+\;p_{+}\;-\;p_{-}\right)p_{-}]}{\partial z}\;+\;\frac{1}{2}\left[p_{+}\;-\;p_{-}\right] \end{array}$$

(35) $$\begin{array}{ccc} De & \;=\; & \frac{t_{r}}{t_{d}}\;=\;{\left(\frac{b_{0}}{2\lambda L_{0}}\right)}^{2} \end{array}$$

where De is the Deborah number, representing the ratio between the relaxation time $t_{r}$ and the characteristic diffusion time $t_{d}\;=\;L_{0}^{2}/D$.

Correspondingly, the Gel–Solvent interface GSI evolves according to the swelling velocity at GGI:

(36) $$\frac{dGSI(t)}{dt}\;=\;-b_{0}\left[p_{+}\left(x,t\right)\;-\;p_{-}\left(x,t\right)\right]{{|}_{GSI(t)}\;⟹\;\frac{dGSI(\tau )}{d\tau }\;=\;-\sqrt{De}\;\left[p_{+}\left(z,\tau \right)\;-\;p_{-}\left(z,\tau \right)\right]|}_{GSI(\tau )}$$

The relaxation time $t_{r}$ also controls the relaxation of the solvent volume fraction ϕ at GSI(t) towards the asymptotic equilibrium value $\phi_{eq}$ [22]. This can be accounted for, in the partial wave formulation, as:

(37) $$t_{r}\frac{dp_{eq(t)}}{dt}\;=\;\phi_{eq}\;-\;p_{eq}\left(t\right)\;⟹\;\frac{dp_{eq}\left(\tau \right)}{d\tau }\;=\;\phi_{eq}\;-\;p_{eq}\left(\tau \right)$$

This implies a time-dependent boundary condition for the partial wave $p_{-}\left(z,\tau \right)$—namely:

(38) $$p_{-}\left(z,\tau \right)\;=\;p_{eq}\left(\tau \right)\;-\;p_{+}\left(z,\tau \right)\;at\;z\;=\;GSI\left(\tau \right)$$

For the partial wave $p_{+}\left(z,\tau \right)$, the boundary condition at the Glass–Gel interface GGI(τ) switches from a Dirichlet boundary condition $p\left(z,\tau \right)\;=\;\phi_{G}$ to an impermeability condition when the Glassy phase disappears—i.e.,:

(39) $$\begin{array}{ccc} p_{+}\left(z,\tau \right) & \;=\; & \phi_{G}\;-\;p_{-}\left(z,\tau \right)\;at\;z\;=\;GGI\left(\tau \right)\;>\;0 \end{array}$$

(40) $$\begin{array}{ccc} p_{+}\left(z,\tau \right) & \;=\; & p_{-}\left(z,\tau \right)\;at\;z\;=\;GGI\left(\tau \right)\;=\;0 \end{array}$$

Meanwhile, the Glass–Gel interface evolves according to the Stefan condition:

(41) $$\left(\phi_{G}\;-\;\phi_{0}\right)\frac{dGGI(t)}{dt}\;=\;J_{p}\;+\;v_{sw}p\;⟹\;\frac{dGGI(\tau )}{d\tau }\;=\;\sqrt{De}\;{\left.\frac{\left[p^{+}\;-\;p_{-}\right]\left[1\;-\;p_{+}\;-\;p_{-}\right]}{\phi_{G}\;-\;\phi_{0}}\right|}_{GGI(\tau )}$$

As for the parabolic transport scheme, the initial conditions for $p_{\pm }\left(z,\tau \right)$, GGI(τ), and GSI(τ) are GGI(0) = 1 − ϵ, GSI(0) = 1, and $p\left(z,0\right)\;=\;\phi_{G}$ that implies for the partial waves $p_{+}\left(z,0\right)\;=\;p_{-}\left(z,0\right)\;=\;\phi_{G}/2$.

This approach can be generalized to include the effect of both a relaxation time $t_{r}\left(p\right)$ and a solvent diffusivity D(p) that are exponential functions of the point-wise solvent volume fraction ϕ(x,t) = p(x,t), as established by simplified versions of the free volume theory [25,26,27]. By setting in Equation (20):

(42) $$\ell \left(p\right)\;=\;\exp\left(\gamma_{r}\;\frac{\phi_{G}\;-\;p}{\phi_{G}\;-\;\phi_{eq}}\right)\;and\;\beta \left(p\right)\;=\;\exp\left(\gamma_{d}\;\frac{\phi_{G}\;-\;p}{\phi_{G}\;-\;\phi_{eq}}\right)$$

the case of an exponentially decreasing relaxation time and an exponentially increasing diffusion coefficient in addressed, i.e.,:

(43) $$t_{r}\left(p\right)\;=\;\frac{1}{2\;{\bar{\lambda }}_{0}}\exp\left(-\gamma_{r}\;\frac{\phi_{G}\;-\;p}{\phi_{G}\;-\;\phi_{eq}}\right)\;,\;D\left(p\right)\;=\;\frac{{\bar{b}}_{0}^{2}}{2\;{\bar{\lambda }}_{0}}\exp\left(\left(2\gamma_{d}\;-\;\gamma_{r}\right)\;\frac{\phi_{G}\;-\;p}{\phi_{G}\;-\;\phi_{eq}}\right)$$

By setting $\gamma_{d}\;=\;\gamma_{r}/2$ the case of an exponentially decreasing relaxation time and a constant diffusion coefficient $D\;=\;D_{0}\;=\;\frac{{\bar{b}}_{0}^{2}}{2\;{\bar{\lambda }}_{0}}$ can be described as well. For both cases, the transport equations for the partial waves and moving boundaries become:

(44) $$\begin{array}{ccc} \frac{\partial p_{+}}{\partial \tau_{0}} & \;=\; & -\sqrt{De_{0}}\;\;\frac{\partial [\beta \left(p\right)\left(1\;-\;p_{+}\;+\;p_{-}\right)p_{+}]}{\partial z}\;-\;\frac{1}{2}\ell \left(p\right)\left[p_{+}\;-\;p_{-}\right] \end{array}$$

(45) $$\begin{array}{ccc} \frac{\partial p_{-}}{\partial \tau_{0}} & \;=\; & \sqrt{De_{0}}\;\;\frac{\partial [\beta \left(p\right)\left(1\;+\;p_{+}\;-\;p_{-}\right)p_{-}]}{\partial z}\;+\;\frac{1}{2}\ell \left(p\right)\left[p_{+}\;-\;p_{-}\right] \end{array}$$

(46) $$\begin{array}{ccc} \frac{dp_{eq}}{d\tau_{0}} & \;=\; & \ell \left(p_{eq}\right)\left[\phi_{eq}\;-\;p_{eq}\left(\tau \right)\right] \end{array}$$

(47) $$\begin{array}{ccc} \frac{dGSI}{d\tau_{0}} & \;=\; & -\sqrt{De_{0}}\;\beta \left(p\right)\left[p_{+}\;-\;p_{-}\right]{|}_{GSI(\tau )} \end{array}$$

(48) $$\begin{array}{ccc} \frac{dGGI}{d\tau_{0}} & \;=\; & \sqrt{De_{0}}\;\beta \left(p\right){\left.\frac{\left[p^{+}\;-\;p_{-}\right]\left[1\;-\;p_{+}\;-\;p_{-}\right]}{\phi_{G}\;-\;\phi_{0}}\right|}_{GGI(\tau )} \end{array}$$

where $\tau_{0}\;=\;2{\bar{\lambda }}_{0}t$ and $De_{0}\;=\;{\left(\frac{{\bar{b}}_{0}}{2{\bar{\lambda }}_{0}L_{0}}\right)}^{2}$ is the Deborah number evaluated at $p\;=\;\phi_{G}$, representing the maximum value of the Deborah number during the course of the swelling process.

## 5. Analysis of Sorption Curves

The numerical solution of the transport scheme Equations (33)–(41) furnishes the spatio-temporal evolution of the partial waves $p_{+}\left(z,\tau \right)$ and $p_{-}\left(z,\tau \right)$, as well as the temporal evolution of the moving interfaces GGI(τ) and GSI(τ) for constant relaxation time $t_{r}$ and solvent diffusivity *D*.

The transport equations for the partial waves $p_{\pm }\left(z,\tau \right)$ have been numerically solved with a home-made Matlab code implementing a finite-difference approach with (i) $N\;=\;5\;\times \;10^{3}$ discretization points for each partial wave, (ii) a second-order upwind representation of convective terms, and (iii) a proper rescaling of the space variable $\tilde{z}\left(\tau \right)\;=\;(\left(z\;-\;GGI\left(\tau \right)\right)/\left(GSI\left(\tau \right)\;-\;GGI\left(\tau \right)\right)$ to transform the moving boundary problem GGI(τ) ≤ z ≤ GSI(τ) into a fixed boundary problem $0\;\leq \;\tilde{z}\left(\tau \right)\;\leq \;1$, ∀τ > 0 [37,38]. The resulting set of 2N + 2 ordinary differential equation has been numerically solved with the multistep solver ode15s with specified relative tolerance $10^{-5}$ and absolute tolerance $10^{-8}$.

**Figure 2:**
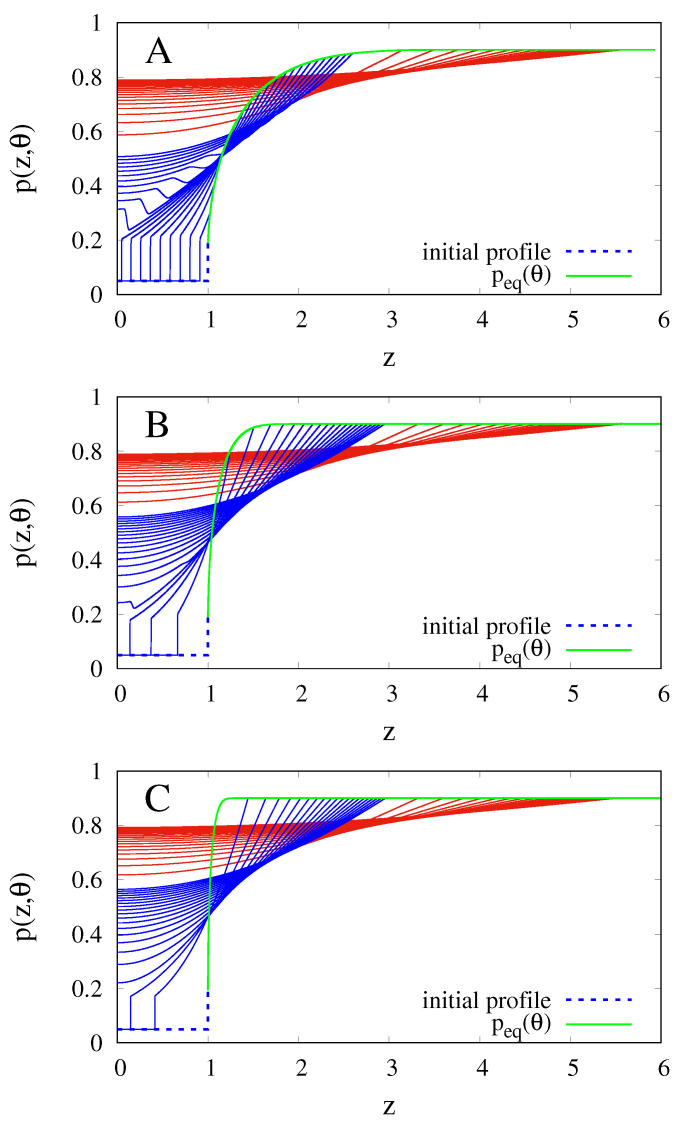
Spatio-temporal evolution of the overall probability density p(z,θ) = ϕ(z,θ) for increasing dimensionless time instants—namely, θ = 0, 0.2, 0.4… up to θ = 2 (blue curves) and θ = 4, 6, 8… up to θ = 40 (red curves). (**A**) De = 1; (**B**) De = 0.1; (**C**) De = 0.01.

Figure 2A–C show the solvent volume fraction profiles p(z,θ) at different time instants during the course of the sorption process, for different values of the Deborah number, De = 1, 0.1, 0.01. If it is assumed to keep constant the solvent diffusivity *D* and the initial dry film thickness $L_{0}$, the dimensionless time $\theta \;=\;t/t_{d}\;=\;tD/L_{0}^{2}\;=\;\tau \;De$ can be introduced and different experiments for different values of De correspond to different polymer relaxation times.

Figure 2A–C show a sharp discontinuity of the solvent concentration at the Glass-Gel interface [17] that moves towards the symmetry axis z = 0. When GGI(θ) reaches the symmetry axis, the discontinuity, for larger values of De—i.e., for larger relaxation times (Figure 2A,B)—is reflected back to the external surface while, for smaller values of De (Figure 2C ) is rapidly smoothed away like in the parabolic case, solution of the transport scheme Equations (22)–(25).

**Figure 3:**
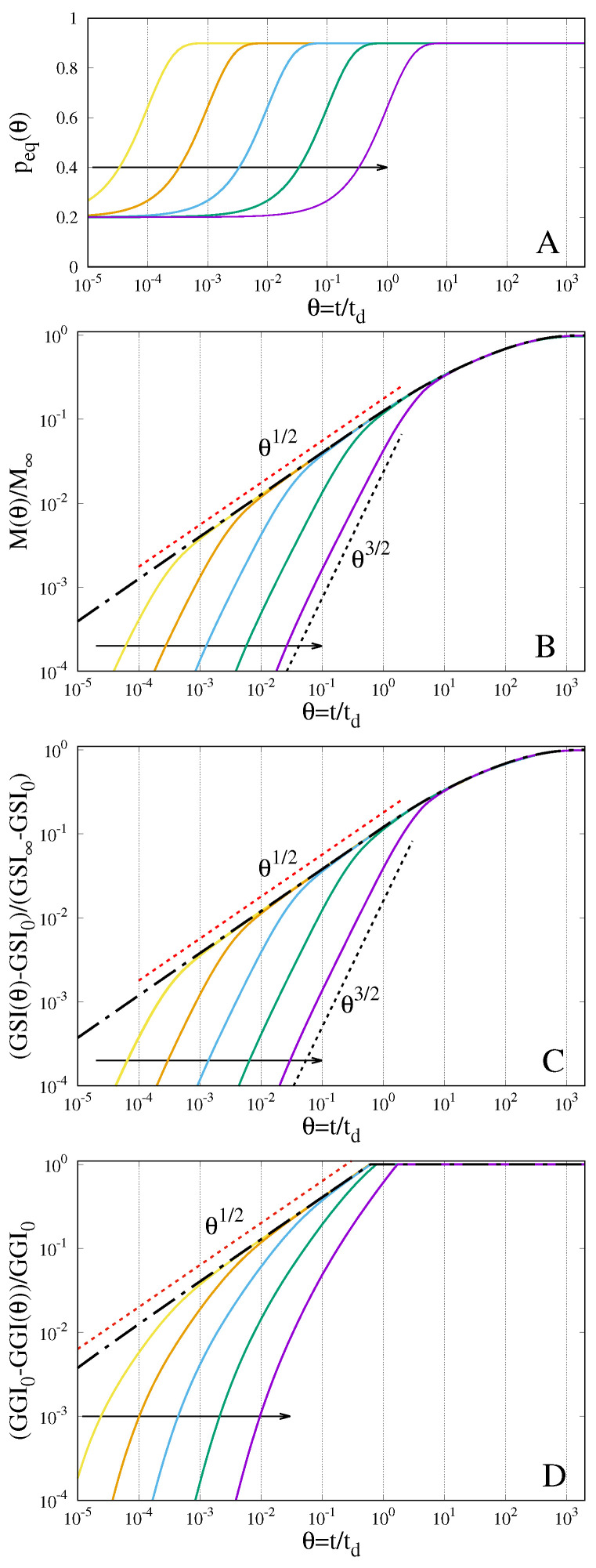
Temporal evolution of the boundary solvent volume fraction $p_{eq}\left(\theta \right)$ (**A**), the normalized amount of absorbed solvent (**B**), the rescaled Gel–Solvent interface (**C**) and the rescaled Glass–Gel interface (**D**). Arrows indicate increasing values of $De\;=\;10^{-4},\;10^{-3},\;10^{-2},\;10^{-1},\;10^{0}$. Dot-dashed black lines represent the behaviour of the parabolic transport scheme, representing the limiting case De → 0.

The comparison between the parabolic and the hyperbolic case is presented in Figure 3B, showing the temporal evolution of the amount of absorbed solvent M(θ), normalized with respect to its equilibrium value $M_{\infty }$, for De → 0 (parabolic case, dot-dashed black curves) and for increasing values of De. It can be observed that, the larger De—i.e., the larger the relaxation time is, the slower the sorption curve that follows an “anomalous” $\theta^{3/2}$ scaling before collapsing onto the sorption curve of the parabolic scheme when the boundary value $p_{eq}\left(\theta \right)$ reaches its asymptotic value $\phi_{eq}$, as shown in Figure 3A. The classical $\theta^{1/2}$ scaling of the standard parabolic scheme represents the envelope of sorption curves only for small-intermediate values of De, namely $De\;\leq \;10^{-2}$. For higher values of De, the sorption curves exhibit a smooth transition from the $\theta^{3/2}$ scaling to the asymptotic saturation behaviour and no $\theta^{1/2}$ scaling can be detected.

A very similar behaviour is observed for the temporal evolution of the Gel–Solvent interface GSI(θ) shown in Figure 3C. Additionally, for the rescaled Gel–Solvent interface, slower dynamics and a net $\theta^{3/2}$ scaling are observed for larger values of De. The larger De, the slower the swelling dynamics, and this also influences the dynamics of the Glass-Gel interface GGI(θ), as shown in Figure 3D. Indeed, for this case the disappearance of the Glass phase requires a longer time.

**Figure 4:**
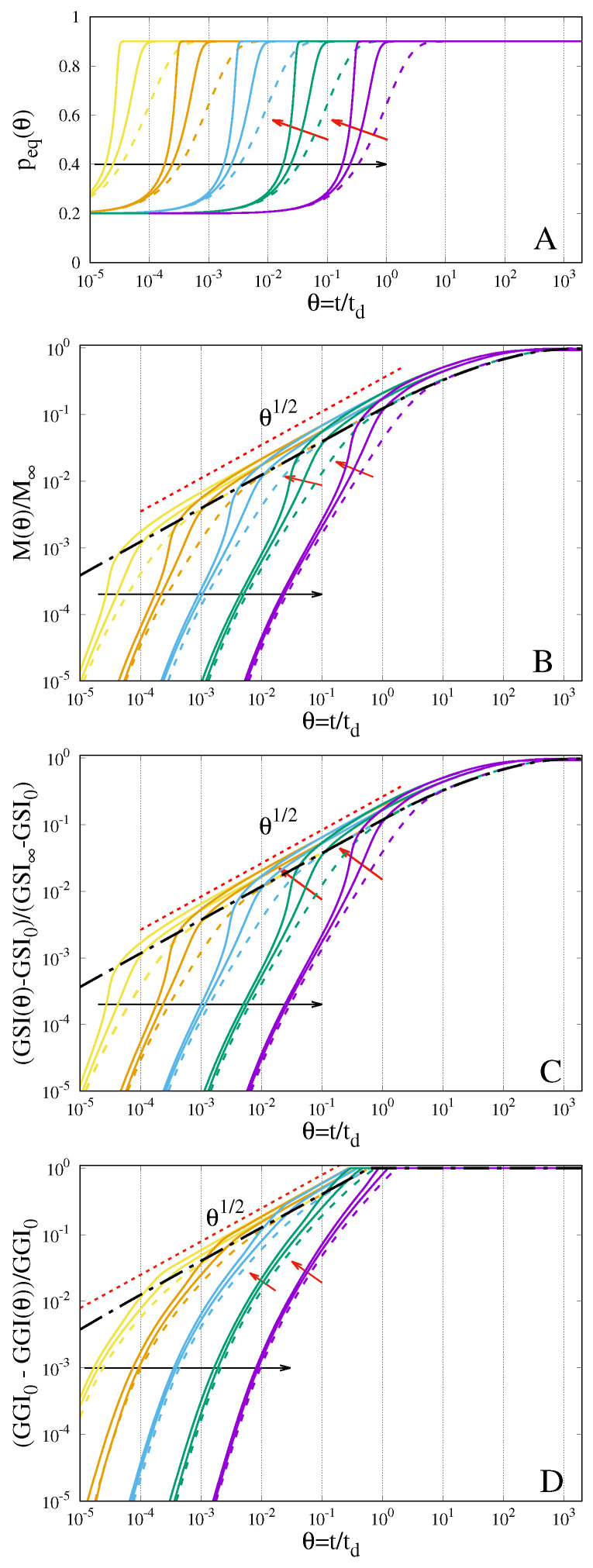
Temporal evolution of the boundary solvent volume fraction $p_{eq}\left(\theta \right)$ (**A**), the normalized amount of absorbed solvent (**B**), the rescaled Gel–Solvent interface (**C**), and the rescaled Glass–Gel interface (**D**). Continuous lines represent the behaviour for a variable relaxation time ($\gamma_{r}\;=\;\log10,\;$log100) and constant diffusion coefficient ($\gamma_{d}\;=\;\gamma_{r}/2$). Dashed lines represent the behaviour for a constant relaxation time and constant diffusivity (same data shown in Figure 3A–D). Larger black arrows indicate increasing values of $De_{0}\;=\;10^{-4},\;10^{-3},\;10^{-2},\;10^{-1},\;10^{0}$. Smaller red arrows indicate increasing values of $\gamma_{r}$.

A more complex behaviour of the swelling system can be observed when the case of a non-constant relaxation time is analyzed. The case of an exponentially decreasing relaxation time $t_{r}\left(p\right)$ and a constant effective solvent diffusivity *D* is here addressed, see Equation (43) with $\gamma_{d}\;=\;\gamma_{r}/2$. The results of the numerical integration of the transport scheme Equations (44)–(48) for $\gamma_{r}\;=\;\log10,\;\log100$ are shown in Figure 4A–D together with the results for a constant relaxation time $\gamma_{r}\;=\;0$, data already shown in Figure 3A–D. Sorption curves for $\gamma_{r}\;>\;0$ and high values of De clearly show good conformity to case II kinetics, feature V in the classification of sorption kinetics by Sanopoulou and Petropoulos [4].

It should be observed that, for $\gamma_{r}\;=\;\log10$, the relaxation time decreases by one order of magnitude from $t_{r}\left(\phi_{G}\right)$ to $t_{r}\left(\phi_{eq}\right)$ while, for $\gamma_{r}\;=\;\log100$, it decreases by two orders of magnitude. It is therefore natural to expect that, the larger $\gamma_{r}$ the faster the sorption dynamics as well as the evolution of the Gel–Solvent interface, as shown in Figure 4B,C. However, it can be readily observed that the sorption curves for $\gamma_{r}\;>\;0$ do not settle down onto the asymptotic behaviour of the parabolic scheme Equations (22)–(25), as in the case of a constant relaxation time $\gamma_{r}\;=\;0$. The same phenomenon is observed for the temporal evolution of the Gel–Solvent interface, as depicted in Figure 4C.

This feature of the sorption process is intrinsically due to the nonlinear convective term, introduced and discussed in Section 3, Equation (21), arising from a characteristic velocity $b_{0}$ and a transition rate λ that are continuous functions of the overall density *p*, as introduced in the Generalized Poisson–Kac process to model a non-constant relaxation time $t_{r}\left(p\right)$. Indeed, in the case under investigation that accounts for the swelling velocity, the Kac limit for the overall density p(z,θ) satisfies the following parabolic equation:

(49) $$\frac{\partial p}{\partial \theta }\;=\;\frac{\partial }{\partial z}\left[\frac{\beta^{2}\left(p\right)}{\ell (p)}\;\frac{\partial p}{\partial z}\left(1\;-\;p\right)\right]\;-\;\frac{\partial }{\partial z}\left[-\frac{\beta (p)}{\ell (p)}\frac{\partial \beta (p)}{\partial p}\frac{\partial p}{\partial z}\left(1\;-\;p\right)\;p\right]$$

By comparing Equation (49) with the corresponding Equation (22) of the parabolic transport model, we can easily recognize the presence of a new convective term, highly nonlinear, characterized by a point-wise negative velocity, that facilitates solvent penetration and speeds up the outward movement of the Gel-Solvent interface. More specifically, in the case of a constant solvent diffusivity ($2\gamma_{d}\;=\;\gamma_{r}$), such as that presented in Figure 4A–D, Equation (49) attains the form:

(50) $$\frac{\partial p}{\partial \theta }\;=\;\frac{\partial }{\partial z}\left[\frac{\partial p}{\partial z}\left(1\;-\;p\right)\right]\;-\;\frac{\gamma_{r}}{2(\phi_{G}\;-\;\phi_{eq})}\frac{\partial }{\partial z}\left[\frac{\partial p}{\partial z}\left(1\;-\;p\right)\;p\right]$$

This convective term (the second term in the right hand side of Equation (50)) can be significant and amplified in interval absorption experiments, where the concentration jump $\left(\phi_{G}\;-\;\phi_{eq}\right)$ can be small. Its presence in the Kac-limit transport equation for the overall density can describe the transition from the S-shaped to the two-stage regime experimentally observed in interval absorption experiments [4].

## 6. Drug Release

This section investigates the influence of the polymer relaxation time on the release kinetic of a drug initially loaded in the thin dry film. In the absence of drug-polymer interaction, drug release in a 1-d swelling system can be simply modeled by a 1-d advection-diffusion equation for the drug concentration $c_{d}\left(x,t\right)$, describing drug transport in the gel layer, along the preferential swelling direction *x*

(51) $$\frac{\partial c_{d}}{\partial t}\;=\;-\frac{\partial J_{d}}{\partial x}\;=\;-\frac{\partial }{\partial x}\left(-D_{d}\frac{\partial c_{d}}{\partial x}\;+\;v_{sw}\;c_{d}\right),\;GGI\left(t\right)\;<\;x\;<\;GSI\left(t\right).$$

where $D_{d}$ is the drug effective diffusivity in the swelling film.

Drug transport is strongly influenced by swelling dynamics not only because of the presence of the swelling convective term but mainly because of the moving boundaries GGI(t) and GSI(t). Specifically, the Glass–Gel interface GGI(t) controls the drug release rate from the Glass to the Gel phase, while the Gel–Solvent interface GSI(t) controls the length of the diffusive path for the drug to be released in the external environment. Indeed, the drug transport Equation (51) must be solved simultaneously with the equations describing the swelling dynamics that furnish, at each time instant, the point-wise swelling velocity $v_{sw}\left(x,t\right)$, as well as the position of the GGI(t) and the GGI(t) interfaces.

The boundary condition adopted for drug concentration $c_{d}\left(x,t\right)$ at the GGI(t) interface is the Stefan condition:

(52) $$\left(c_{d}\;-\;c_{d}^{0}\right)\;\frac{dGGI(t)}{dt}\;=\;J_{d}\;\;at\;x\;=\;GGI\left(t\right)$$

where $c_{d}^{0}$ is the initial drug concentration, supposed uniform in the dry film. A perfect sink condition at the Gel–Solvent interface—i.e., $c_{d}{|}_{GSI(t)}\;=\;0$—is assumed without loss of generality.

The total amount of drug (per unit surface area) $M^{d}\left(t\right)$ released up to time *t* can be evaluated as:

(53) $$M^{d}\left(t\right)\;=\;2\int_{0}^{t}D_{d}\frac{\partial c_{d}}{\partial x}{|}_{GSI(t^{\prime })}dt^{\prime }\;=\;2\left(c_{d}^{0}L_{0}\;-\;\int_{GGI(t)}^{GSI(t)}c_{d}\left(x^{\prime },t\right)dx^{\prime }\right)$$

Correspondingly, given the perfect sink condition adopted, the total amount of drug released at equilibrium is $M_{\infty }^{d}\;=\;2\;c_{d}^{0}L_{0}$, equal to the total amount of drug initially loaded in the dry film.

For the sake of simplicity, here we analyze the case of a constant drug diffusion coefficient $D_{d}$. The swelling velocity $v_{sw}$ and the temporal evolution of the GGI and GSI interfaces can be obtained from the solution of the parabolic transport scheme Equations (22)–(25) corresponding to De → 0 or from the solution of the hyperbolic transport scheme Equations (33)–(41) for different values of the Deborah number, corresponding to different polymer relaxation times for a constant solvent diffusivity *D*. A new parameter α needs to be introduced, representing the drug to solvent diffusivity ratio $\alpha \;=\;D_{d}/D$.

**Figure 5:**
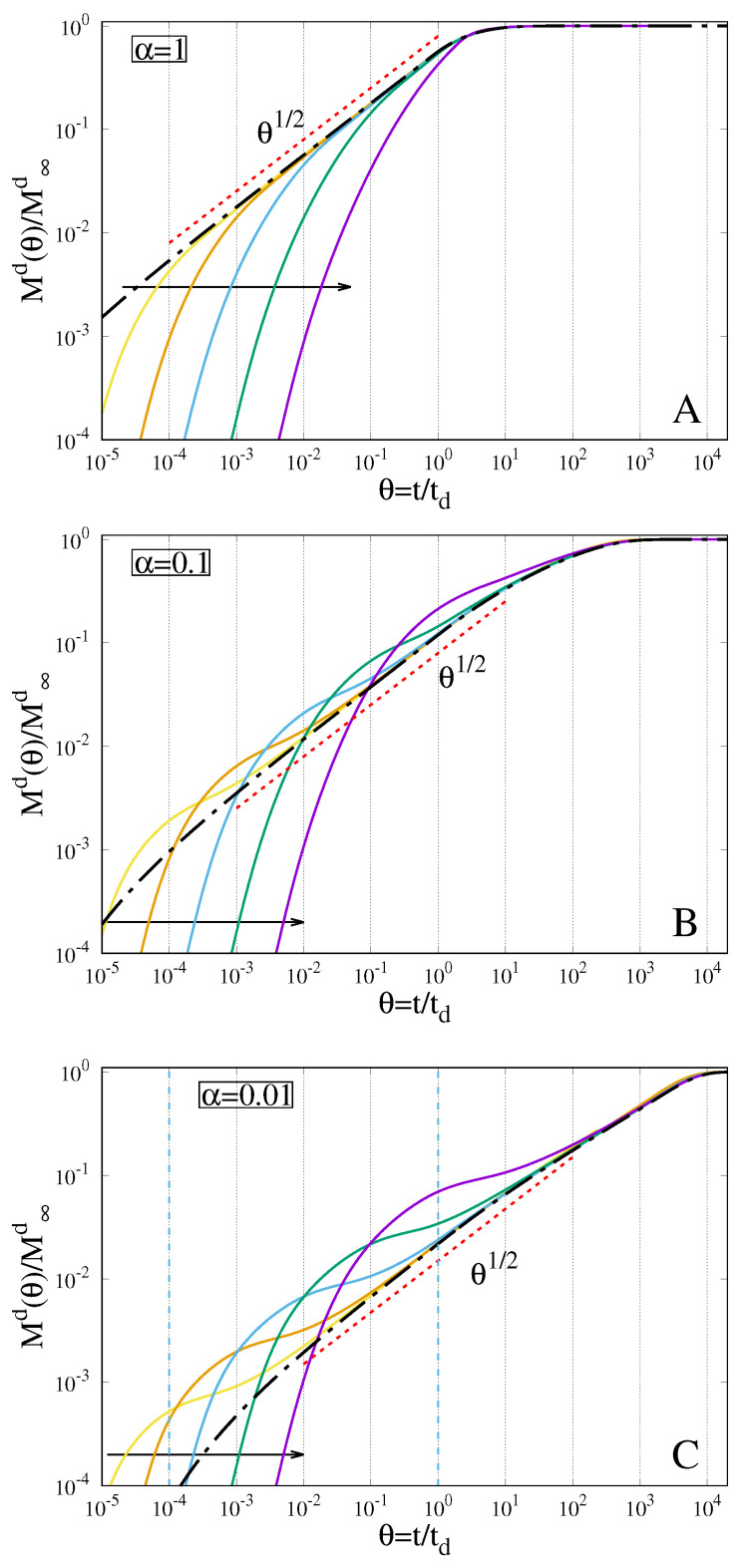
Drug release curves $M^{d}\left(\theta \right)/M_{\infty }^{d}$ for different values of De and $\alpha \;=\;D_{d}/D$. Arrows indicate increasing values of $De\;=\;10^{-4},\;10^{-3},\;10^{-2},\;10^{-1},\;10^{0}$. Dot-dashed black lines represent the limiting case De → 0. (**A**) α = 1; (**B**) α = 0.1; (**C**) α = 0.01.

Figure 5A–C show drug release curves for α = 1, 0.1, 0.01. When drug and solvent diffusivity are of the same order, i.e., α = 1 (Figure 5A), drug release curves for De > 0 approach monotonically the drug release curve for De = 0. The larger De, the slower the release and the release curve for De > 0 is always below (slower than) the release curve for De = 0.

This behaviour significantly changes when the drug diffusivity is one order (α = 0.1) or two orders of magnitude (α = 0.01) smaller than solvent diffusivity. Indeed, Figure 5B,C clearly show that, when α = 0.1, the drug release curves for De > 0, for intermediate time scales, become faster and approach “from above” the release curve for De = 0. This effect is amplified in intensity and time duration for the smaller value α = 0.01 analyzed.

In order to explain this phenomenon, we need to consider that drug release is strongly influenced by the movement of the two interfaces GGI and GSI. In particular, the Gel–Solvent interface controls the length of the drug diffusive path. The larger De, the slower the GSI velocity at short-intermediate time scale, the shorter the diffusive path and consequently the faster the release kinetics. Moreover, the smaller the drug diffusivity, the more important the effect of a shorter diffusive path on the release.

**Figure 6:**
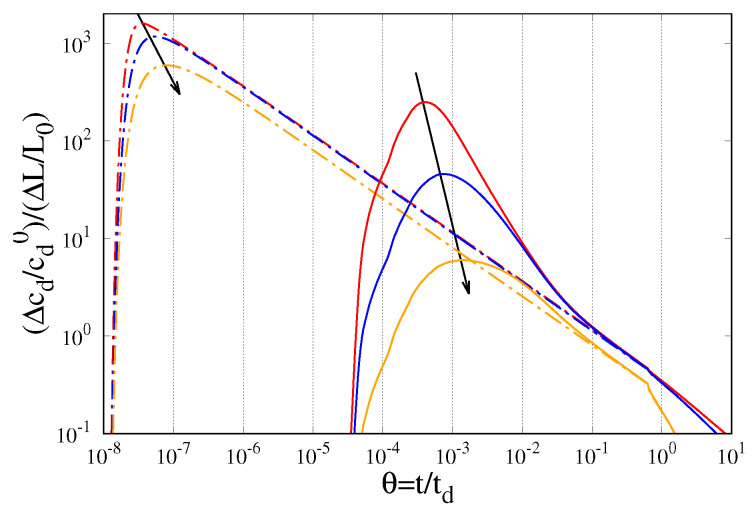
Dimensionless macroscopic drug concentration gradient $\left(\Delta c_{d}/\Delta L\right)\left(L_{0}/c_{d}^{0}\right)$ vs θ for De = 0 (dashed lines) and for $De\;=\;10^{-2}$ (continuous lines). Arrows indicate increasing values of $\alpha \;=\;D_{d}/D\;=\;0.01,\;0.1,\;1$.

A shortcut estimation of the drug flux at the outer boundary GSI can be obtained from the macroscopic concentration gradient:

(54) $$-D_{d}{\left.\frac{\partial c_{d}}{\partial x}\right|}_{GSI}\;\propto \;\frac{\Delta c_{d}}{\Delta L}\;=\;\frac{c_{d}{{|}_{GGI}\;-\;c_{d}|}_{GSI}}{GSI\;-\;GGI}\;=\;\frac{c_{d}{|}_{GGI}}{GSI\;-\;GGI}$$

shown in Figure 6 for the three values of α analyzed. Dashed lines represent the temporal evolution of the dimensionless macroscopic concentration gradient $\frac{\Delta c_{d}/c_{d}^{0}}{\Delta L/L_{0}}$ for De = 0, while continuous lines represent the same quantity for $De\;=\;10^{-2}$. It can be readily observed that, for α = 0.01, the macroscopic concentration gradient for $De\;=\;10^{-2}$ is significantly larger than that for De = 0 (red curves) exactly in the time interval $10^{-4}\;\leq \;\theta \;\leq \;10^{-2}$ during which there is an “inversion” of the release curves (see Figure 5C, azure curve). This effect is still present but less intense for α = 0.1 (blue curves) and actually disappears for α = 1 (orange curves), in agreement with the observed behaviour of the release kinetics shown in Figure 5A,B.

**Figure 7:**
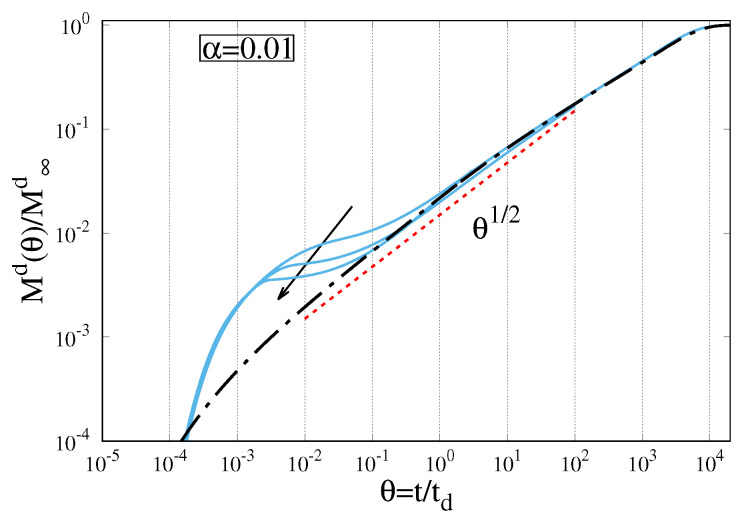
Drug release curves $M^{d}\left(\theta \right)/M_{\infty }^{d}$ for $De\;=\;10^{-2}$, $\alpha \;=\;D_{d}/D\;=\;0.01$, $\gamma_{d}\;=\;\gamma_{r}/2$ and $\gamma_{r}\;=\;0,\;\log10,\;\log100$. The arrow indicates increasing values of $\gamma_{r}$. The dot-dashed black line represents the limiting case De → 0.

As a confirmation of this explanation, Figure 7 shows the behaviour of the drug release kinetics for a polymer relaxation time that is an exponentially decreasing function of the solvent volume fraction. In this case, the faster movement of the Gel–Solvent interface for De > 0 and $\gamma_{r}\;>\;0$, already presented and discussed in Section 5, implies a faster increase in the drug diffusion path and a consequent slow down of the release kinetics. Indeed, Figure 7 shows that the larger $\gamma_{r}$, the smaller the overshoot of the release curve with a consequent faster collapse onto the parabolic release curve (De = 0).

## 7. Conclusions

The article investigates the influence of polymer relaxation time on sorption curves and drug release kinetics. Case II diffusion processes are addresses and analyzed through the theory of Poisson–Kac stochastic processes possessing finite propagation velocity.

The article provides a first, physically significant application of Poisson–Kac processes to moving-boundary problems associated with the swelling dynamics in polymeric matrices. Moreover, it corresponds to a physically relevant example in which the parameters of the process depend in a nonlinear way on the concentration of the diffusing species. This case has been treated theoretically in [13] by considering simple models of exclusion processes. Here, it finds a practically relevant application related to transport in polymeric systems. Moreover, the way of handling boundary conditions represents a generalization of the analysis developed in [39].

A preliminary analysis of sorption curves for a constant polymer relaxation time clearly shows that the larger the Deborah number De, the slower the sorption curve characterized by an anomalous $\theta^{3/2}$ short–intermediate time-scale behaviour. The classical $\theta^{1/2}$ scaling of the standard parabolic scheme represents the envelope of sorption curves only for small–intermediate values of $De\;\leq \;10^{-2}$ while, for higher values of De, the sorption curve exhibits a smooth transition from the $\theta^{3/2}$ scaling to the asymptotic saturation behaviour.

A more complex and unexpected behaviour of the sorption curves has been observed when the case of an exponentially decreasing relaxation time is analyzed. Indeed, sorption curves for De > 0 are, as expected, slower than the corresponding sorption curve for De = 0 at short time-scales. However, in longer time-scales, there is an inversion and the sorption process becomes faster for De > 0 than for De = 0. This phenomenon is intrinsically due a nonlinear convective term, arising from the introduction of a characteristic velocity and a transition rate that are continuous functions of the overall density. This convective term, quantified in Equation (50), facilitates solvent penetration and speeds up the outward movement of the Gel–Solvent interface.

An equally unexpected behaviour has been observed for the drug release curves, when the influence of polymer relaxation time on release kinetics is investigated. Indeed, when drug diffusivity is one order or two orders of magnitude smaller than the solvent diffusivity, the role of polymer relaxation time (De > 0) is to speed up the drug release kinetics at a small–intermediate time scale, with the process being controlled by a smaller length of the diffusive path for the drug to be released in the external environment.

All these observations are extremely important in the analysis of experimental data of sorption and drug release curves and can reasonably explain many of the “anomalous” behaviours that cannot be described by employing the more classical parabolic transport schemes. The model does not introduce new fitting parameters with respect to those introduced in other hyperbolic models and can be applied in a wide range of Deborah numbers $De\in [10^{-4}–10^{3}]$ without any stability issues for the numerical solution of the partial waves transport equations. Obviously, being a mechanistic model described by partial differential equations, its application to the analysis of experimental transient sorption data requires the numerical integration of the transport equations and the use of basic optimization tools for the best fit of transport parameters. A similar procedure can be found in [40], where the rate-type viscoelastic model of Camera-Roda and Sarti [21] is applied to reproduce the anomalous sorption of fluoropolymer-solvent systems.

What is relevant in tracing the connection between hyperbolic transport theory and stochastic processes is that the model equations used in this article are suitable for a direct stochastic description, meaning that all the results obtained in this article can in principle be obtained from the stochastic Lagrangian kinematics of solute particles. Within the scientific community interested in transport properties in polymeric systems, the stochastic interpretation of the constitutive equations with memory associated with the glassy–rubbery transition and with the influence of viscoelasticity to mass transport is missing. This article provides a rational way to fill this gap.

The PK approach can be naturally extended to two/three dimensional problems as well as to include more than one distinct structural relaxation process with different relaxation times. 

## Data Availability

The data presented in this study are available on request from the corresponding author.
